# The effect of the partnership between DanceSport couples on competitive performance: the mediating role of athlete engagement

**DOI:** 10.3389/fpsyg.2023.1278874

**Published:** 2023-11-16

**Authors:** Xiuxia Liu, Bo Wu, Xinghe Weng, Qi Shan

**Affiliations:** ^1^Department of Physical Education, Xiamen University, Xiamen, China; ^2^Department of Physical Education, Beijing Technology and Business University, Beijing, China; ^3^Department of Health and Physical Education, The Education University of Hong Kong, Tai Po, Hong Kong SAR, China

**Keywords:** DanceSport, partnership, competitive performance, athlete engagement, self-determination theory

## Abstract

**Objectives:**

Although the positive association of partnership between DanceSport couples with competitive performance (CP) is documented, less is known about the mediating factors of this relationship. According to the related literature and self-determination theory (SDT), the present study finds and verifies that athlete engagement (AE) mediates the association between partnership and competitive performance.

**Methods:**

A total of 242 Chinese sports dancers were recruited using the purposive sampling method. The Partnership Scale-DanceSport Couples (PS-DSC), the Athlete Engagement Questionnaire (AEQ), and the Competitive Performance Questionnaire (CPQ) were adopted to collect data.

**Results:**

The obligatory instrumental ties, expressive ties, and interpersonal perception scores are all positively correlated with both athlete engagement and competitive performance, and athlete engagement scores are positively correlated with competitive performance. Athlete engagement completely mediates the association between obligatory instrumental ties and competitive performance, and it partially mediates the association between expressive ties, interpersonal perception, and competitive performance, with the mediating effect accounting for 25.29 and 24.40% of the total effect, respectively.

**Conclusion:**

Athlete engagement mediates the association between DanceSport couples’ partnership and competitive performance. High levels of athlete engagement are needed to improve the chance of promoting obligatory instrumental ties, expressive ties, and interpersonal perception between DanceSport couples toward excellent competitive performance. Overall, the results represent an attempt to extend our understanding of the mechanisms by which the three partnership stereotype factors individually influence dancers’ cognitive and psychological states.

## 1. Introduction

Achieving high-level competitive performance is important for competitive sports (Taylor et al., 2022). To improve competitive performance, researchers have traditionally focused on individual-level variables such as anxiety (Hardy and Parfitt, 1991). However, Isoahola (1995) proposes an interactionist model, which indicates that behavior or performance is a function of the interaction between the person and their environment [*B* = *f* (P X E)]. Interpersonal relationship, in this context, is a very important environment for athletes to achieve excellent competitive results, which is supported by the literature on the coaches-athletes relationship (Jowett and Meek, 2000; Jowett and Cockerill, 2003; Jowett and Ntoumanis, 2004; Jowett and Poczwardowski, 2007; Jowett and Palmer, 2010; Davis et al., 2013) and the athlete-athlete partnership (Poczwardowski et al., 2019). As a competitive sport, DanceSport requires partners to follow the rhythm of the music and compete against a couple of contestants to display the beauty of the sport (Chae and Koh, 2012). Therefore, competitive performance is also affected by the partnership between DanceSport couples (e.g., Fostiak, 1996; Majoross et al., 2008; Lai, 2014; Budnik-Przybylska et al., 2015). Even the top dancers in the world are not immune to the fluctuations in their performance caused by problems in dance partnerships. For example, Slavik Kryklyvyy, the legendary Ukrainian dancer, kept changing partners, resulting in a lower world ranking. Similarly, Ralf Lepehne, Germany’s top dancer, retired early after his partner split.

The partnership between DanceSport couples is the psychological projection of mutual adaptation and the basis of high-level cooperation (Majoross et al., 2008). The premise of showing a perfect image in the competition is to establish a stable and high-quality partnership (Fostiak, 1996). Scholars find that partners who achieve elite performance are always in a romantic partnership (Pistole, 2003; Ifrar et al., 2020). The reason may be that the nature of the partnership affects the performance of partner skills, which is one of the important judging factors in the World DanceSport Federation competitions (Remelcˇ et al., 2019; Yoshida et al., 2020). During the competition, judges look for body control, posture, shape, footwork, timing, rhythm, and the level of difficulty of the routine (Pittman et al., 2005), and they assess all these components within a noticeably fleeting period. With up to 50 couples on the floor in the early heats, judges eliminate 50% of the couples in 2 min (Tremayne and Ballinger, 2008). Therefore, it is not enough to execute technically correct steps but to make it look effortless, graceful, enjoyable, and harmonious. Even as in other sports that combine esthetic art with athletic ability, due to the subjective nature of scoring, the judgment may lead to unexpected or undesired results, which is still maintained for now (Tremayne and Ballinger, 2008).

Given that partnership is so vital, any analysis of the various problems surrounding DanceSport always considers the partnership between couples (Wiesława et al., 2013). Therefore, belittling or ignoring partnerships might hurt performance (e.g., persistence, motivation, and success) (Davis et al., 2013). However, thus far, there are few theories to guide the management methods of partnership. The reason may be that the mediating factor of the association of partnership between DanceSport couples with competitive performance remains unclear. Therefore, to address this problem, in this study, we draw useful experiences from the previous research results. Based on the conceptual framework of the partnership between DanceSport couples (expressive ties, obligatory instrumental ties, and interpersonal perception) (Liu et al., 2022; 2023), we review the literature on the relationship between partnership and competitive performance and propose athlete engagement as an important mediating variable based on self-determination theory and other research findings. In addition, the first author of our study has been involved in professional DanceSport training for seven years and has competition experience; she has a deep sense and understanding of partnerships, which is also extremely valuable in completing this study.

### 1.1. Partnership between DanceSport couples and competitive performance

#### 1.1.1. Obligatory instrumental ties and competitive performance

Obligatory instrumental ties (OIT) refer to the reciprocal behavior tendency of elite dance couples based on the principle of obligation ruled by *“renqing (favor) or mianzi (face*).” It mixes instrumental and obligatory factors (Liu et al., 2023), which is also possessed by elite dancers. On the one hand, achieving excellent competitive performance is rewarded with international recognition; on the other hand, dancers who are unable to achieve excellent competitive performance are eliminated in the elimination rounds (Joanna, 2015, p. 26). Therefore, to obtain prizes and prestige, dancers take the competitive victory as the most important starting point for participation (Budnik-Przybylska et al., 2015), and partners are always considered instrumental tools and assets that help dancers improve their athletic ability and performance outcomes (Lai, 2014). Consequently, partners often exhibit a high degree of reciprocity and interaction (Reisman, 1981; Marion, 2006), and the partnership is taken as an instrument to obtain benefits (Lai, 2014; Liu et al., 2023). Even when describing the qualities of an ideal dance partner, a good dancer states that a perfect partner is one who helps them improve their dancing skills and stand out in competitions (Marion, 2006). Therefore, the questionnaire on obligatory instrumental ties includes the following four items: “Cooperating with my partner promotes my ability,” “Cooperating with my partner will make me grow professionally,” “My partner and I are tied together,” and “Cooperating with my partner will get me closer to my goal” (Liu et al., 2023). These items all reflect the obligatory instrumental ties between DanceSport partners in order to obtain excellent competition results.

On the other hand, there is a strong emphasis on obligation ruled by *“renqing”* between partners, which is different from the contractual obligation emphasizing taking an interest. In Asian countries, such as China, the obligation between DanceSport couples is stressed (Liu et al., 2023). This obligation is defined by *“renqing”* or *“mianzi”* based on Confucianism, which is based on personal feelings rather than commercial law to uphold the obligation. When people fail to follow the rules, they invite public criticism. So, they obey the rules to maintain harmonious relationships (Hwang, 1987; Seligman, 1999). Thus, the obligatory instrumental ties questionnaire developed by Liu et al. (2023) includes the item: “X20 I obligately follow the training plan agreed with my partner,” indicating that dance partners must obey the training plan to dance together, which is vital to achieving better performance.

#### 1.1.2. Expressive ties and competitive performance

Expressive ties refer to the emotional bond between elite dance couples (Liu et al., 2023), which exerts a profound influence on dancers’ partnerships (Julia, 2011, pp. xii-46; Majoross et al., 2008). It includes instant intimacy (Julia, 2011, pp. 20–21) and long-term affection (Brewińska and Poczwardowski, 2012; Yang, 2015). Instant intimacy is a short-term enthusiastic state of desire formed between dance partners in the context of the competition. Long-term affection develops over a long period of time, resulting from interactions in personal and professional contexts, which is different from instant intimacy marked by weaker emotional concentrations and slower emotional outbursts. For long-term affection to be established, dancers must feel appreciated and cared for and be in a harmonious relationship with their partners.

Based on the previous literature, the expressive ties between DanceSport couples have a positive impact on competitive performance (e.g., Fostiak, 1996; Majoross et al., 2008; Brewińska and Poczwardowski, 2012; Lai, 2014; Park and Choi, 2014; Budnik-Przybylska et al., 2015; Liu et al., 2023). In particular, studies involving participants in dyadic groups have found that dyadic groups perform better if the members like each other (Krivonos et al., 1976). Very often, elite DanceSport couples are also pairs in life (Majoross et al., 2008; Brewińska and Poczwardowski, 2012), and the connection between them is intimate, passionate, and immersive (Yang, 2015).

The reasons are as follows: first, DanceSport is a discipline based on romantic fantasies about love and sex between heterosexual dyads (Julia, 2011, pp. xii). It requires open expression of sexual intimacy (Julia, 2011; Harman, 2019), and romance is at the core of the sport (John, 1998, p. 11). Therefore, the instant intimacy among the couples in competition is a key factor in achieving excellence. Although sexual attraction does not pass between the dancing couples (McMains, 2006; Gainor, 2007; Joanna, 2015, p. 61), most of the high-level participants tend to interpret the romantic relationship among the sexes in dance as the passion for their partners, at least in their on-stage performances (Liu et al., 2023). In addition, the idea that instant intimacy is a requirement for partners under mirror neural mechanisms is also supported by neuroscientific perspectives. For example, Fogassi (2011) and Ye et al. (2016) hold that the human cerebral cortex has a “mapping” function of mirror neurons in the inferior parietal lobe, ventral premotor cortical area, and posterior inferior frontal gyrus. This mapping function translates sensual movements, such as tightly connected crotches, closely attracted eyes and breath, and feelings of passion into emotional boosters between them and their partners. Scholars describe sexy movements in detail:

“Dancers employ a number of signals of varying subtlety to express romantic interest within a dance. They include placing hands on a partner’s intimate body parts (such as low hips and buttocks), wrapping arms completely around a partner to ensure shoulder-to-knee contact, stroking a partner’s hair or face, dancing literally cheek to cheek, looking directly into one another’s eyes, and other idiosyncratic gestures” Joanna (2015, p. 61).

“He approaches her from behind until his chest touches her back, then thrusts his hands to her lower thighs and caresses her upwards. After grabbing her waist, he pushes her away and sharply pulls her back to him, provoking an impact of her back against his ribcage” Valentin (2020).

“Shimmering in a beaded gold costume, Tanya alternately approached and fled from Edward, spiraling in toward him, hesitating, smiling, then spinning rapidly away. Hands caressed lightly, fleetingly, arms swirled in a serpentine embrace. Edward moved deliberately to showcase Tanya, their bodies forming luxurious and strangely balletic lines as she acquiesced to his touch. And all the while, their hips undulated in Latin motion, that sensuous pelvic movement that is the essence of the rumba/bolero. Desire begot desire, stirring reveries” Peters (1992).

“The judges watched as the dancers dipped low to the floor, the pelvis wed to the pelvis, man guiding woman into an “over sway.” The dancers moved cautiously, pulling themselves across the floor heel first. One couple had a decidedly Argentine look. Sleek in black, they revolved warily, crouched into the knees, Alicia exaggeratedly arched, Miguel proudly upright. They maintained this posture even as they punctuated their stalking with legs that flashed with lightning speed, hooking around hips and thrusting between thighs. Both executed the sharp libidinous jabs and pelvic climbs, which sometimes began or ended with the elegance of around de jambe. This eroticism vaguely recalled the knives carried by male dancers a century ago in Argentina as they challenged one another to combat Mack the Knife as a tango dancer. In Boston, the judges were not impressed” Peters (1992).

Peters (1992) wonders whether dancers sometimes get caught up in the desire for each other on the dance floor.

Second, long-term affection is a prerequisite for satisfactory performance. To be more specific, excellent competitive performance requires a prolonged period of systematic and professional practice (Ericsson et al., 1993). Like any skill, dancing requires practice—perhaps 5 h for every lesson (John, 1998, p. 168), and dancers may train with a partner for more than 10 or 20 years, or even a lifetime, as John (1998) stressed, “there is no final destination. Learning to dance is a lifetime process, which represents part of its fascination. There will always be a new dance to learn, a new figure to experiment with, and a new dancing style to explore (p. 166).” During this time, they often leave home to train elsewhere, and a rapport between the dancers is essential, whether it is a romantic attraction to each other or a shared passion for dance (John, 1998, p. 27). Eventually, a pattern of harmonious partnership develops, in which partners face difficulties together and share intimate emotions, such as a romantic relationship. Most couples in the field of international professional sports dancers are married (John, 1998, p. 117) and rarely define their partnerships as mere working relationships (Majoross et al., 2008).

While the positive effect of expressive ties between DanceSport couples on competitive performance has been recognized, no study has tested this idea with the widest possible range of investigations, which is the goal of our study.

#### 1.1.3. Interpersonal perception and competitive performance

Interpersonal perception (IP) refers to the ability of elite dance couples to share and expose each other in the full process of taking competition as the goal, to sensitively perceive the psychological and behavioral tendencies of dance partners, including revealing their hearts, sharing, and understanding each other (Liu et al., 2023). Interpersonal perception plays a significant role in improving the quality of training and competitive performance. The reasons may be that interpersonal ability is the engine of artistic communication, and the desire to dance comes from the wish to communicate and feel connected to partners (Dan, 2012). Strengthening the sense of bonding and communication between partners increases the effectiveness of training and satisfaction with competitive performance (Jin and In, 2006). Wulff (1998) flags how “dancers are, for example, extremely skilled at communicating without looking at each other, which is something they learn in dancing but carry over to how they move and behave when they are not dancing” (p. 108).

In addition, the DanceSport partnership is a special bond that mixes expressive ties and instrumental professional ties (Wang, 2018) and falls under the principles of “need” and “fairness,” respectively (Hwang, 1987). Under the principle of “fairness” in expressive ties with partners, cooperation is likely to intersect with love, leading to quarrels and conflicts (Liu et al., 2023). However, under the principle of “need” in instrumental professional ties, dancers may miss training or competitions because of a lack of professionalism. For Chinese dancers, intimacy is about blurring the boundaries between the individual and others so as to achieve a state of separation between “you” and “me” (Lynn, 1998; Sara, 2005). Therefore, we believe that high-quality interpersonal perception can help alleviate the above contradictions. However, this view has not been proven. This study aims to test this view.

### 1.2. Mediating variable: athlete engagement

At present, few studies have explored the mediating variables between partnership and competitive performance, making it difficult to obtain reliable hypotheses from relevant studies on partnerships between DanceSport couples. Therefore, our study draws on the interdependence theory, which forms the basis of the coach-athlete relationship. We constructed this theory by conducting in-depth interviews with four couples. We also explored the pathway of influence that this theory suggests on competitive performance.

Related studies and self-determination theory (SDT) support the hypothesis that athlete engagement mediates the association between partnership and competitive performance. To be more specific, Jowett and Cockerill (2003) show that high-quality communication and respect between athletes and coaches contribute to athletes’ satisfaction and improve competitive performance. Building on his previous study, Jowett and Poczwardowski (2007) propose an integrated research model of the coach-athlete relationship that further clarifies that the relationship between the two affects competitive performance (Jowett and Palmer, 2010). The self-determination theory suggests that a sense of human relatedness and a basic human psychological need is significantly correlated with athlete engagement (Lonsdale et al., 2007; Hodge et al., 2009). Following that, Wang et al. (2014) find that the coach-athlete relationship significantly influences the athlete engagement. They construct a chain mediation model in which athlete engagement is the mediating variable between the coaches-athletes relationship and the satisfaction of competition performance (as a proxy variable of competition performance) (β = 0.04, *p* < 0.001) (Ye et al., 2016).

Based on this literature, it is believed that athlete engagement mediates the association between obligatory instrumental ties, expressive ties, and interpersonal perception, and competitive performance. To be more specific:

1. Athlete engagement between obligatory instrumental ties and competitive performance. Sport dancers need to consciously observe the normative manners with their partners, including mutual respect, appreciation, and a sense of responsibility (especially the responsibility to adhere to the training schedule and to work hard). According to John (1998, p. 33), successful DanceSport competitors are dedicated athletes; the dancers need to devote as much time as possible to practice skill techniques with their partners and be more enthusiastic and dedicated. At the same time, according to Ostrom (2003), repetitive interaction helps individuals to promote mutual benefit and mutual assistance with others. Through continuous cooperative training, partnerships between DanceSport couples generate mutually beneficial behaviors that promote the achievement of the dancers’ own training goals. Furthermore, better-performing dancers tend to be more diligent with a firm belief in their success, confident in attaining their goals, and more motivated (Ifrar et al., 2020). Therefore, to a considerable extent, athlete engagement mediates the association of obligatory instrumental ties with competitive performance.
2. Athlete engagement between expressive ties and competitive performance. A long-term relationship between partners triggers positive psychological feelings, such as recognition among both partners, thereby increasing their athlete engagement. More importantly, through the “mapping” function of mirror neurons in several areas of the human cerebral cortex, the inferior parietal lobule, the ventral premotor gyrus Broca’s area, and the posterior inferior frontal gyrus (Fogassi, 2011; Ye et al., 2016), the tightly absorbed eyes and breath, emotions and feelings between partners get transformed into emotional boosters for the dancers themselves and their partners, which may enhance the infectiousness and competitive performance of the dance. At the same time, the instant intimacy between the partners stimulates the dancers’ passion for the dance and deepens their immersion in the dance (Yang, 2016). This immersion is like vigor and enthusiasm (two dimensions of athlete engagement). Vigor is defined as “a feeling of being physically and mentally active,” and enthusiasm is characterized as “a feeling of excitement and high enjoyment.” Therefore, high-quality expressive ties directly contribute to athlete engagement. This positive psychological trait also enables dancers to overcome difficulties and burnout, thus increasing their competitive ability (Gustafsson et al., 2011). In addition, from the judges’ point of view, they will give the final decision based on whether the partners are harmonious, vigorous, and confident (Tremayne and Ballinger, 2008). Therefore, athlete engagement mediates the association of expressive ties with competitive performance.
3. Athlete engagement between interpersonal perception and competitive performance.

Relatedness is an innate and indispensable element for humans (Ryan, 1995; Deci and Ryan, 2002, 2004), and all dualistic relationships begin with a process of interpersonal perception in which both parties participate (Kang and Bodenhausen, 2015). Therefore, the partnership perception among DanceSport dancers is inevitable, and its satisfaction contributes to the generation of self-determination, motivation, and the maintenance of partnership behavior (i.e., athlete engagement), thus affecting competitive performance. Effective communication and understanding between partners, along with providing encouragement and confidence in difficult situations, are important means to improve compatibility, according to Majoross et al. (2008). When dancers perceive that their partners sincerely express emotional support and reciprocate, they tend to communicate with each other in a more friendly manner and exhibit positive emotional tendencies and behaviors, which becomes instrumental to achieving their expected goals (Miller, 1990). In addition, individuals predict their future behavior through what they know about others in interpersonal interactions (Sang, 2014). Therefore, the higher the degree of interpersonal perception, the more it can provide a good foundation for cooperation with partners and the more it is instrumental in improving the dancers’ athlete engagement and obtaining excellent competitive results. Therefore, athlete engagement mediates the association of interpersonal perception with competitive performance.

### 1.3. The current study: development of theoretical framework and hypotheses

Analyzing DanceSport practices and related theories, we find that athlete engagement mediates the partnership between DanceSport couples (the three dimensions of obligatory instrumental ties, expressive ties, and interpersonal perception) and competitive performance. However, of the studies we reviewed, none had tested the hypotheses with surveys. Hence, the present study aims to test the following hypotheses:

- *H1*: Athlete engagement partially mediates the association between obligatory instrumental ties and competitive performance.
- *H2*: Athlete engagement partially mediates the association between expressive ties and competitive performance.
- *H3*: Athlete engagement partially mediates the association between interpersonal perception and competitive performance.

In addition, since athletic ability also plays a crucial role in competitive performance, it is used as a control variable in this study.

## 2. Methods

### 2.1. Participants

Our sample consisted of 242 participants (see Table 1) who had participated in the 2019 Chinese DanceSport Championship (Beijing Station)—the highest-level event of Chinese DanceSport held at the Ditan Gymnasium. They were selected by five experienced national-level DanceSport judges who were engaged in front-line teaching of DanceSport and were from Beijing Sports University, Wuhan Sports University, Capital University of Physical Education and Sports, Xi’an Physical Education University, and the Institute of Psychology of the Chinese Academy of Sciences (CAS). Among them, there were two professors, two doctors, and one champion. The champion had 20 years of training and experience in DanceSport, was the champion of Latin dance among the Chinese professional team for three consecutive years (2013–2016), and broke international competition records among Chinese athletes. The judges used the following selection criteria: (a) having a regular partner for at least 3 years; (b) outstanding performance in the past.

**Table 1:** Demographic information of the participants (*n* = 242).

Variable	Classification	Frequency	Percent(%)
Sex	Male	122	50.4
	Female	120	49.6
Training time	5.1 ~ 10 years	107	44.2
	10.1 ~ 15 years	28	9.92
	>15 years	16	6.61
Partner time	0 ~ 12 month	151	62.4
	13 ~ 36 month	58	24
	37 ~ 60 month	22	9.1
	>60 month	11	4.5
Athletic ability	High-level	134	55.4
	Low-level	108	44.6

### 2.2. Materials

#### 2.2.1. Partnership quality

We used the Partnership Scale-DanceSport Couples (PS-DSC) (Liu et al., 2023), which had a three-dimensional scale consisting of 13 items. Cronbach’s α of the subscales relating to obligatory instrumental ties, expressive ties, and interpersonal perception was 0.905, 0.846, and 0.848, respectively, which established that the scale could be applied in this study.

#### 2.2.2. Athlete engagement

We adopted the Athlete Engagement Questionnaire (AEQ) (Lonsdale et al., 2007), which already had good adaptability among Chinese athletes and has been verified (Wang et al., 2014; Ye, 2014; Ye et al., 2016). The scale with its 16 items had Cronbach’s α of 0.951, and its four dimensions had a Cronbach’s α of 0.921, 0.939, 0.900, and 0.873, respectively, proving that the scale could be applied in this study.

#### 2.2.3. Competitive performance

Competitive performance includes the satisfaction degree of field performance and field performance; therefore, we used the Competitive Performance Questionnaire with four items (Liu et al., 2023). Furthermore, the Athlete Satisfaction Questionnaire (ASQ) (Riemer and Chelladurai, 1998) was adopted. The competition ranking was determined by the performance outcomes, which were evaluated by five international-level DanceSport judges. The ranking was assigned to a 5-level Likert scale, which took into account the five levels of ordinal variables. The results obtained after processing the data from these variables were considered unbiased, even when combined with continuous variables. The scale consisted of four items, and Cronbach’s α was 0.704, which indicated that the scale could be applied in this study.

### 2.3. Analysis strategy

We used SPSS 22.0 to process and analyze the data. First, the Pearson correlation was used to examine the relationship between partnership, athlete engagement, and competitive performance. Second, all variables were standardized, and then Model 4 in PROCESS, developed by Hayes (2018), was adopted to test the mediating effect of athlete engagement between partnership and competitive performance, controlling for gender, training time, partner time, and athletic ability.

### 2.4. Common method variance bias

Since the data used in this study was collected from self-report questionnaires, common method variance bias (CMVB), which affects the results and even draws wrong conclusions, was a potential concern. The possibility of CMVB is assessed when independent and dependent variables are measured under the same context and obtained from the same source. We adopted the harmony one-factor test to inspect whether there was an artificial covariation between the independent and dependent variables. The results showed that the variance of the first common factor explanation was 18.05%, which is lower than the 40% criterion proposed by Podsakoff et al. (2003). Therefore, CMVB was not found in this study.

## 3. Results

As shown in Table 2, obligatory instrumental ties, expressive ties, and interpersonal perception scores were all positively correlated with athlete engagement and competitive performance. Furthermore, athlete engagement scores were positively correlated with competitive performance.

**Table 2:** Correlation matrix of each research variable.

	OIT	ET	IP	AE	CP
OIT	1				
ET	0.58**	1			
IP	0.55**	0.67**	1		
AE	0.59**	0.40**	0.35**	1	
CP	0.19**	0.37**	0.37**	0.36**	1

** indicates *p* < 0.01; OIT: obligatory instrumental ties; ET: expressive ties; IP: interpersonal perception. AE: athlete engagement and CP: competitive performance.

After controlling for gender, training time, partner time, and athletic ability, three regression models were established to analyze the influence of partnership quality on competitive performance through the moderating effect of athlete engagement. In the first model, obligatory instrumental ties were used as the independent variable. The model and regression coefficient are shown in Table 3.

**Table 3:** Results of the mediating effects of athlete engagement between obligatory instrumental ties and competitive performance.

Dependent variable	Independent variable	β	SE	*t*	95% Bootstrap CL		*R*^2^	*F*
					LLCI	ULCI		
CP	Gender	−0.01	0.12	−1.65	−0.44	0.04	0.16	8.75
	Training time	0.07	0.07	1.11	−0.06	0.22		
	Partner time	0.22	0.07	3.49**	0.11	0.40		
	Athletic ability	0.19	0.12	3.18**	0.15	0.63		
	OIT	0.12	0.06	2.01*	0.00	0.25		
AE	Gender	−0.14	0.11	−2.46*	−0.50	−0.06	0.27	17.82
	Training time	−0.05	0.07	−0.82	−0.19	0.08		
	Partner time	−0.01	0.07	−0.16	−0.15	0.12		
	Athletic ability	0.10	0.11	1.68	−0.03	0.41		
	OIT	0.48	0.06	8.45***	0.37	0.60		
CP	Gender	−0.06	0.12	−0.92	−0.336	0.12	0.24	12.17
	Training time	0.09	0.07	1.42	−0.04	0.24		
	Partner time	0.22	0.07	3.71***	0.12	0.40		
	Athletic ability	0.16	0.12	2.78**	0.10	0.55		
	OIT	−0.04	0.07	−0.56	−0.17	0.10		
	AE	0.33	0.07	4.99***	0.20	0.47		

* indicates *p* < 0.05 **, *p* < 0.01; and ***, *p* < 0.001. CP: competitive performance; AE: athlete engagement; and OIT: obligatory instrumental ties.

As seen in Table 3, obligatory instrumental ties significantly influenced competitive performance (*β* = 0.12, *t* = 2.01, p<0.05) when athlete engagement was added to the regression model. Obligatory instrumental ties by itself could not significantly influence competitive performance (*β* = −0.04, *t* = −0.56, *p* > 0.05); however, it could significantly influence athlete engagement (*β* = 0.48, *t* = 8.45, p<0.001) and athlete engagement could influence competitive performance (*β* = 0.33, *t* = 4.99, p<0.001). This demonstrated that athlete engagement completely mediated the effect of obligatory instrumental ties on competitive performance. Thus, H1 that athlete engagement partially mediates the association between obligatory instrumental ties and competitive performance is supported by this study.

In the second model, expressive ties were used as the independent variable. The model and regression coefficient are shown in Table 4.

**Table 4:** Results of the mediating effects of athlete engagement between expressive ties and competitive performance.

Dependent variable	Independent variable	β	SE	*t*	95% Bootstrap CL		*R*^2^	*F*
					LLCI	ULCI		
CP	Gender	−0.05	0.12	−0.87	−0.33	0.13	0.25	15.66
	Training time	0.03	0.07	0.45	−0.11	0.17		
	Partner time	0.18	0.07	2.98**	0.07	0.35		
	Athletic ability	0.25	0.12	4.23***	0.26	0.72		
	ET	0.35	0.06	5.81***	0.23	0.47		
AE	Gender	−0.12	0.12	−2.03*	−0.47	−0.01	0.20	11.77
	Training time	−0.05	0.07	−0.74	−0.19	0.09		
	Partner time	−0.02	0.07	−0.25	−0.16	0.12		
	Athletic ability	0.16	0.12	2.65**	0.08	0.55		
	ET	0.41	0.06	6.54***	0.28	0.53		
CP	Gender	−0.02	0.11	−0.42	−0.27	0.18	0.29	15.75
	Training time	0.04	0.07	0.62	−0.09	0.18		
	Partner time	0.18	0.07	3.11**	0.08	0.35		
	Athletic ability	0.21	0.12	3.67***	0.20	0.65		
	ET	0.26	0.06	4.09***	0.14	0.39		
	AE	0.22	0.06	3.52**	0.10	0.34		

* indicates *p* < 0.05; **, *p* < 0.01; and ***, *p* < 0.001. CP: competitive performance; AE: athlete engagement; and ET: expressive ties.

As seen in Table 4, expressive ties significantly influenced competitive performance (*β* = 0.35, *t* = 5.81, p<0.001) when athlete engagement was added to the regression model. Expressive ties could also independently significantly influence competitive performance (*β* = 0.26, *t* = 4.09, *p* < 0.001) and athlete engagement (*β* = 0.41, *t* = 6.54, *p* < 0.001), and athlete engagement could significantly influence competitive performance (*β* = 0.22, *t* = 3.52, *p* < 0.01). The direct effect value of expressive ties affecting competitive performance was 0.26 [95% confidence intervals (CI) = 0.14, 0.39]. In addition, athlete engagement partially mediated the effect of expressive ties on competitive performance [indirect effect = 0.09, 95% CI = 0.04, 0.15]. The direct effect value and indirect value accounted for 74.71 and 25.29% of the total effect, respectively. Thus, H2 proposing that athlete engagement partially mediates the association between expressive ties and competitive performance is supported by this study.

In the third model, interpersonal perception was used as the independent variable. The model and regression coefficient are shown in Table 5.

**Table 5:** Results of the mediating effects of athlete engagement between interpersonal perception and competitive performance.

Dependent variable	Independent variable	β	SE	*t*	95% Bootstrap CL		*R*^2^	*F*
					LLCI	ULCI		
CP	Gender	−0.06	0.12	−1.07	−0.35	0.10	0.24	14.95
	Training time	0.01	0.07	0.22	−0.12	0.15		
	Partner time	0.18	0.07	2.95**	0.07	0.35		
	Athletic ability	0.23	0.12	4.02***	0.24	0.69		
	IP	0.34	0.06	5.54***	0.22	0.46		
AE	Gender	−0.14	0.12	−2.32*	−0.52	−0.04	0.16	9.18
	Training time	−0.05	0.07	−0.82	−0.21	0.09		
	Partner time	−0.01	0.07	−0.15	−0.16	0.14		
	Athletic ability	0.14	0.12	2.30*	0.041	0.52		
	IP	0.35	0.06	5.53***	0.226	0.48		
CP	Gender	−0.03	0.11	−0.52	−0.28	0.16	0.29	15.69
	Training time	0.03	0.07	0.43	−0.11	0.16		
	Partner time	0.18	0.07	3.08**	0.08	0.35		
	Athletic ability	0.20	0.11	3.51**	0.18	0.62		
	IP	0.25	0.06	4.05***	0.13	0.38		
	AE	0.23	0.06	3.87***	0.11	0.35		

* indicates *p* < 0.05; **, *p* < 0.01; and ***, *p* < 0.001. CP: competitive performance; AE: athlete engagement; and IP: interpersonal perception.

As seen in Table 5, interpersonal perception significantly influenced competitive performance (*β* = 0.34, *t* = 5.54, *p* < 0.001) when athlete engagement was added to the regression model. Interpersonal perception could also significantly influence competitive performance (*β* = 0.25, *t* = 4.05, *p* < 0.001) and athlete engagement (*β* = 0.35, *t* = 5.53, *p* < 0.001), and athlete engagement could significantly influence competitive performance (*β* = 0.23, *t* = 3.87, *p* < 0.001). The direct effect value of interpersonal perception affecting competitive performance was 0.25 [95% CI = 0.13, 0.38]. In addition, athlete engagement partially mediated the effect of interpersonal perception on competitive performance [indirect effect = 0.08, 95% CI = 0.03, 0.14]. The direct effect value and indirect value accounted for 75.60 and 24.40% of the total effect, respectively. Thus, H3 on athlete engagement partially mediating the association between interpersonal perception and competitive performance is supported by this study.

## 4. Discussion

Our results indicate that athlete engagement completely mediates the association between obligatory instrumental ties and competitive performance (H1), partially mediates the association between expressive ties and competitive performance (H2), and partially mediates the association between interpersonal perception and competitive performance (H3). These findings demonstrate that obligatory instrumental ties, expressive ties, and interpersonal perception between DanceSport couples and athlete engagement are critical factors influencing individuals’ competitive performance. The findings are a result of the self-determination theory (SDT) and research mentioned in the current study’s introduction and other theories.

### 4.1. Athlete engagement completely mediates the association between obligatory instrumental ties and competitive performance

In our study, athlete engagement completely mediates the association between obligatory instrumental ties and competitive performance. It suggests that athlete engagement influences the relationship between obligatory instrumental ties and competitive performance. On its own, obligatory instrumental ties are unable to significantly influence competitive performance, contradicting the hypothesis of economic man. Moreover, it also contradicts the theory of group dynamics, which holds that a harmonious interpersonal atmosphere within a group can stimulate the creation of excellent competitive performance in a sports team. For dance dyads, their partnership is premised on meeting specific needs and achieving competitive goals. The reason may lie in special norms inherent in Chinese culture relating to instrumental relationships, which weaken the utilitarian purpose and pay more attention to “renqing” and “mianzi” (Hwang, 1987). Establishing and maintaining interpersonal relationships is to maintain an interpersonal relationship that is different from other partnerships that emphasize mission and work completion, and the latter is covered under the principle of “contractual obligations.” For Chinese dancers, showing utilitarian tendencies in their interpersonal relationships with their partners is not supported by “renqing” and “mianzi” and can even undermine the stability of partnerships, the quality of athlete engagement, and their impact on performance outcomes. In a previous study, when interviewed by our first author, a DanceSport champion said, “We are like a business relationship,” embarrassing his partner on the spot. The partnership later broke apart, with each of them finding new partners (Liu et al., 2023). During follow-up interviews aimed at understanding the reason for the partner splitting, it was found that the interpersonal relationship was also filled with a more exposed utilitarian purpose. Therefore, the study holds that instrumental ties should foreground the Chinese “obligatory” principle, such as “renqing” and “mianzi,” that considers partnership as not only a tool to achieve personal goals but also to maintain and develop partnerships. By maintaining the partnership between dance couples, dancers can promote each other’s dedication, enthusiasm, and confidence and promote athlete engagement. In this way, obligatory instrumental ties can promote performance results.

### 4.2. Athlete engagement partially mediates the association between expressive ties and competitive performance

Our results indicate that athlete engagement partially mediates the association between expressive ties and competitive performance (H2). This finding demonstrates that expressive ties and athlete engagement are critical factors influencing individuals’ competitive performance, which also reaffirms findings under relevant literature (Julia, 2011, pp. xii-46; Majoross et al., 2008). On the one hand, instant intimacy—a vital element of expressive ties referred to as a short-duration passionate state of desire formed between dance partners in the competition context—can showcase romantic sexual relationships and give a good impression to the judges. The reason may be that the inferior parietal lobule of the human cerebral cortex, the Broca region before the ventral motor, and the posterior part of the inferior frontal gyrus have the “mapping” function of mirror neurons (Fogassi, 2011; Ye et al., 2016). This mapping function turns sexy movements such as intricately connected crotch, closely attracted eyes and breath, and passionate feelings into an emotional booster between the partners, transforming “interpretation” into “experience,” at least a real experience on the court, potentially further enhancing the appeal and expressiveness of dance. In addition, the instant intimacy (e.g., passion) of the dancers deepens their immersion in the dance (Yang, 2016), which becomes the sufficient basis for judges to give “marks (scores)” (Tremayne and Ballinger, 2008). Based on the passionate movements, Peters (1992) points out that even dancers get confused by the desire expressed by partners on the dance floor and are surprised whether they are just a partner off the court.

On the other hand, expressive ties, including long-term affection, which requires the dancers to appreciate, care for, and be harmonious with their partners, refers to an emotional tie generated in long-time interactions in life and professional contexts between dance partners. According to Krivonos et al.’s (1976) research on the social-psychological attributes of interpersonal relationships between two people shows that, if dancers like each other, they will perform better. The care, appreciation, and other emotions formed by the long time of the dance partners will help them to complete the competition routine of showing the intimate partnership among the sexes with a more open mind, help stimulate positive feelings, and promote the dancers’ athlete engagement and competitive performance.

### 4.3. Athlete engagement partially mediates the association between interpersonal perception and competitive performance

Our results indicate that athlete engagement partially mediates the association between interpersonal perception and competitive performance (H3). This finding demonstrates that interpersonal perception between DanceSport couples and athlete engagement is a critical factor that influences individuals’ competitive performance. This finding is not only proven under SDT and its applied research but is also supported by many psychological theories. According to Kang and Bodenhausen (2015), all dyadic relationships begin with a personal perception process in which both parties participate. The satisfaction of the dancers’ sense of partnership helps in motivating self-determination, thus promoting athlete engagement and competitive performance. In addition, individuals can predict their future behavior by understanding others in interpersonal interactions. Increased communication and understanding between dance partners, tolerance toward each other, and offering more encouragement and confidence to dance partners in difficult situations are important means to improve tacit cooperation (Majoross et al., 2008).

Social exchange theory and Pavlov’s dynamic stereotype theory also support our results. To be more specific, according to social exchange theory, when dancers perceive that their sense of unfamiliarity with their partners has diminished and instead perceive sincere and expressive ties between them, they tend to communicate in a more friendly manner, often displaying positive emotional tendencies. This can lead to behaviors that are beneficial for achieving their desired goals. On the contrary, the performance (such as persistence and success) of athletes can be affected if the partnership is belittled or ignored (e.g., coach-athlete relationship) (Isoahola, 1995). Therefore, DanceSport partners can promote the satisfaction of interpersonal perception by being open with each other by engaging with and treating each other sincerely, stimulating the cooperative behavior of continuous cooperation and effort. Likewise, according to Pavlov’s dynamic stereotype theory, after repeated practice, the dance movement can be shaped, and the excitement and inhibition of the cerebral cortex are more concentrated and accurate in time and space. It is not only helpful for accurate and beautiful movements but can also control and complete the movements unconsciously. So only when dancer partners reach a certain tacit understanding can they pay more attention to the overall artistic expression, which is the key to obtaining competitive performance (Majoross et al., 2008; Liu et al., 2023), often among elite dancers (Majoross et al., 2008). In addition, it needs to be emphasized that within Chinese culture, the state of mutual understanding, mutual benefit, and heart-to-heart connection is to ease the blurring of the “I-He” boundary of intimate relationships and inhibit the contradiction in the athletes’ rational thinking and field theory that requires the athletes to rationally deal with emotional and cooperative issues to ensure effective training and improve competitive performance. To sum up, having a high degree of interpersonal perception leads to a better foundation for cooperation among dance couples, and it also promotes athlete engagement, resulting in excellent competitive performance.

## 5. Conclusion

This study found that based on relationship-related theories, such as SDT and Pavlov’s dynamic stereotype theory, athlete engagement mediates the association between obligatory instrumental ties, expressive ties, interpersonal perception, and competitive performance. Among them, the intermediary effect of athlete engagement explains 100% of the variation of obligatory instrumental ties. It partially mediates between expressive ties, interpersonal perception, and competitive performance, respectively, with the upper and lower limits of the 95% confidence interval of the mediating effect being (0.036, 0.148) and (0.034, 0.138). The effect values of 0.088 and 0.082 accounted for 25.29 and 24.40% of the total effect. Overall, the results represent an attempt to extend our understanding of the mechanisms by which partnerships influence dancers’ cognitive and psychological states.

In addition, the results apply to countries influenced by Confucian culture, such as South Korea and Japan, and apply to professional athletes rather than amateur athletes.

## 6. Strengths and limitations

The findings of this study should be considered in light of two strengths and two limitations. The two strengths include: 1) offering a path for transforming DanceSport partnerships into competitive performances and providing dancers with high-quality ideas for strengthening partnerships; 2) Further strengthening the scientific nature of the self-determination theory and providing evidence to enrich and improve the theory of self-determination to a certain extent. Nonetheless, due to the preliminary nature of our study, two limitations should also be noted. First, the sample comprised dance couples only from China. This has implications for the international generalizability of the study findings. Future research with diverse samples from other countries should be considered. Second, the sample size needs to be expanded further. Because of the need to control as much as possible the influence of referees, lighting, venues, and other factors on the results of the study, we obtained data from a single match. However, it is difficult to increase the sample size beyond 242 people in a single match. In the future, other methods need to be explored to compensate for the difficulty of expanding the sample size so as to avoid statistical bias in the study.

## 7. Future research directions

DanceSport couples are cognitively, emotionally, and behaviorally interdependent. However, the two have gender and individual differences, and how their psychology and behavior affect each other needs to be further studied. Our study suggests that, in the future, the actor-partner interdependence mediation model (methods of dyadic data analysis for interpersonal relationships between couples) can be used to further explore the relationship between partnerships, athlete engagement, and competitive performance. Thus, opinions can be provided to male and female dancers, respectively, to ensure excellent competitive performance.

## Data availability statement

The raw data supporting the conclusions of this article will be made available by the authors, without undue reservation.

## Ethics statement

The studies involving humans were approved by the Ethics Committee of the Wuhan Sport University. The studies were conducted in accordance with the local legislation and institutional requirements. The participants provided their written informed consent to participate in this study.

## Author contributions

XL: Conceptualization, Data curation, Formal analysis, Investigation, Methodology, Project administration, Resources, Software, Supervision, Validation, Visualization, Writing – original draft, Writing – review & editing. BW: Conceptualization, Funding acquisition, Writing – original draft. XW: Funding acquisition, Methodology, Writing – review & editing. QS: Writing – review & editing.

## References

[ref1] Brewińska A. Poczwardowski A. (2012). “Exploration of positive elements in the sporting ballroom dancing couples’interpersonal relations: the results of qualitative research” in Optimization of sports and health training from the perspective of psychology. eds. Blecharz J. Siekańska M. Tokarz A. (Cracow: Academy of Physical Education im. Bronisława Czech Republic)

[ref2] Budnik-Przybylska D. Lewandowska-Walter A. Czyz˙yk P. (2015). Subjective and objective determinants of the satisfaction from the cooperation in a dance couple. Health Psychol. Rep. 3, 35–46. doi: 10.5114/hpr.2015.48989

[ref9003] Chae J. S. Koh Y. B. (2012). The Relationship between Perfectionism, Partnership, and Sport Coping in Dancesport Players. J. Sport and Lei. Stud. 49, 387–399.

[ref3] Dan V. (2012). Artistic communication and dance sport particularities. Proc. Soc. Behav. Sci. 46, 4869–4873. doi: 10.1016/j.sbspro.2012.06.351

[ref4] Davis L. Jowett S. Lafrenière M.-A. K. (2013). An attachment theory perspective in the examination of relational processes associated with coach-athlete dyads. J. Sport Exerc. Psychol. 35, 156–167. doi: 10.1123/jsep.35.2.156, PMID: 23535974

[ref6] Deci E. L. Ryan R. M. (2004). Intrinsic motivation and self-determination in human behavior. Encycl. Appl. Psychol. 3, 437–448. doi: 10.1016/B0-12-657410-3/00689-9

[ref5] Deci E. L. Ryan R. M. (2002). “Self-determination research: reflections and future directions” in Handbook of self-determination research. eds. Deci E. L. Ryan R. M. (Rochester: University of Rochester Press)

[ref7] Ericsson K. A. Krampe R. T. Tesch-Römer C. (1993). The role of deliberate practice in the acquisition of expert performance. Psychol. Rev. 100, 363–406. doi: 10.1037/0033-295x.100.3.363

[ref8] Fogassi L. (2011). The mirror neuron system: how cognitive functions emerge from motor organization. J. Econ. Behav. Organ. 77, 66–75. doi: 10.1016/j.jebo.2010.04.009

[ref9] Fostiak D. (1996). Motor coordination of rhythmic gymnastics athletes, figure. Skating athletes and ballroom dancing athletes. Gdańsk: Wydawnictwo Uczelniane AWF.

[ref10] Gainor Ellen. (2007).“‘But who leads?‘same-sex ballroom dance and the heteronormative paradigm.” Paper presented at the Annual Meeting of the Association of Theater in Higher Education, New Orleans.

[ref11] Gustafsson H. Hassmén P. Hassmén N. (2011). Are athletes burning out with passion? Eur. J. Sport Sci. 11, 387–395. doi: 10.1080/17461391.2010.536573

[ref13] Hardy L. Parfitt G. (1991). A catastrophe model of anxiety and performance. Br. J. Psychol. 82, 163–178. doi: 10.1111/j.2044-8295.1991.tb02391.x 1873650

[ref14] Harman V. (2019). The sexual politics of ballroom dancing. London: Springer Nature Limited.

[ref15] Hayes A. F. (2018). Introduction to mediation, moderation, and conditional process analysis: A regression-based approach. New York: Guilfor Press.

[ref16] Hodge K. Lonsdale C. Jackson S. A. (2009). Athlete engagement in elite sport: an exploratory investigation of antecedents and consequences. Sport Psychol. 23, 186–202. doi: 10.1123/tsp.23.2.186

[ref17] Hwang K. K. (1987). Face and favor: the Chinese power game. Am. J. Sociol. 92, 944–974. doi: 10.1086/228588

[ref18] Ifrar T. Majoranc K. Kajtna T. (2020). Matching of personality trains, emotional intelligence and social skills among dance partners in competitive dancing. Kinesiology 52, 242–249. doi: 10.26582/k.52.2.9

[ref19] Isoahola S. E. (1995). Intrapersonal and interpersonal factors in athletic performance. Scand. J. Med. Sci. Spor. 5, 191–199. doi: 10.1111/j.1600-0838.1995.tb00035.x, PMID: 7552764

[ref20] Jin H. M. In H. K. (2006). The difference of partner reliance, team commitment, and Unity in the individual and environment variables of DanceSport expert. J. Sport Leis. Stud. 27, 439–450.

[ref21] Joanna B. (2015). Becoming beautiful: Ballroom dance in the American heartland. Illinois: The Board Trustees of the University of Illinois.

[ref22] John L. R.. (1998). Ballroom dancing: The romance, Thythm and style. San Antonio: Laurel Glen.

[ref23] Jowett S. Cockerill I. M. (2003). Olympic Medallists' perspective of the athlete–coach relationship. Psychol. Sport Exerc. 4, 313–331. doi: 10.1016/s1469-0292(02)00011-0

[ref24] Jowett S. Meek G. A. (2000). The coach-athlete relationship in married couples: an exploratory content analysis. Sport Psychol. 14, 157–175. doi: 10.1123/tsp.14.2.157

[ref25] Jowett S. Ntoumanis N. (2004). The coach-athlete relationship questionnaire (CART-Q): development and initial validation. Scand. J. Med. Sci. Spor. 14, 245–257. doi: 10.1111/j.1600-0838.2003.00338.x, PMID: 15265147

[ref26] Jowett S. Palmer A. (2010). Our understanding the role and significance of a key two-person relationship in sport and executive coaching. Sport Exerc. Psychol. Rev. 6, 19–30. doi: 10.53841/bpssepr.2010.6.2.19

[ref27] Jowett S. Poczwardowski A. (2007). “Understanding the coach-athlete relationship” in Social psychology in sport. eds. Jowette S. Lavallee D. (Champaign, Human kinetics)

[ref28] Julia A. E. (2011). Dance with me: ballroom dancing and the promise of instant intimacy. New York University Press, 20–25.

[ref29] Kang S. K. Bodenhausen G. V. (2015). Multiple identities in social perception, and interaction: challenges and opportunities. Annu. Rev. Psychol. 66, 547–574. doi: 10.1146/annurev-psych-010814-015025, PMID: 25061671

[ref30] Krivonos P. D. Byrne D. Friedrich G. W. (1976). The effect of attitude similarity on task performance. J. Appl. Soc. Psychol. 6, 307–313. doi: 10.1111/j.1559-1816.1976.tb02406.x

[ref31] Lai Y.-T.. (2014). Exploration of the dance partner relationship in dance sport. Taiwan: Asia University.

[ref32] Liu X. X. Wang X. F. (2022). The study of Partnership in Chinese DanceSport couples. Int. Conf. Sports Sci. AESA 6:31.

[ref33] Liu X. X. Yang G. Wang S. Wang X. F. Wang X. L. (2023). Development and initial validation of the partnership scale-DanceSport couples. Front. Psychol. 14, 1032767–1032780. doi: 10.3389/fpsyg.2023.1032767, PMID: 36910793 PMC9995879

[ref34] Lonsdale C. Hodge K. Jackson S. A. (2007). Athlete engagement: II. Development and initial validation of the athlete engagement questionnaire. Int. J. Sport Psychol. 26, 769–785. doi: 10.1016/j.humov.2007.07.007

[ref35] Lynn J.. (1998). Intimacy: Person relationships in modern societies. Cambridge: Cambridge Polity Press.

[ref36] Majoross K. Hamar P. Dózsa I. Dancs H. (2008). The relationship of couples in competitive dancing. J. Hum. Sport Exerc. 3, 12–24. doi: 10.4100/jhse.2008.32.02

[ref37] Marion J. S. (2006). Dance as self, culture, and community: The construction of personal and collective meaning and identity in competitive ballroom and salsa dancing. San Diego, CA: University of California.

[ref38] McMains J.. (2006). Glamour addiction: Inside the American ballroom dance industry. Middletown, Conn: Wesleyan University Press.

[ref39] Miller L. (1990). Intimacy and liking: mutual influence and the role of unique relationships. J. Pers. Soc. Psychol. 59, 50–60. doi: 10.1037/0022-3514.59.1.50, PMID: 2213489

[ref40] Ostrom E. (2003). “Toward a behavioral theory linking trust, reciprocity, and reputation” in Trust and reciprocity; interdisciplinary lessons from experimental research. eds. Ostrom E. Walker J. (New York: Russell Sage Foundation)

[ref41] Park C.-h. Choi J.-h. (2014). The relationship among partnership, perfectionism, and team Performance’s satisfaction in DanceSport players. Kor. Soc. Well. 9, 29–38.

[ref42] Peters S. (1992). The elegant passion. J. Pop. Cult. 25, 163–172. doi: 10.1111/j.0022-3840.1992.1674153.x

[ref43] Pistole C. M. (2003). Dance as a metaphor: complexities and extensions in psychotherapy. Psychother. Theory Res. Pract. 40, 232–241. doi: 10.1037/0033-3204.40.3.232

[ref44] Pittman A. Waller M. Dark C. (2005). Dance a while: A handbook of folk, square, contra, and social dance (9th). New York: Pearson, Benjamin Cummings.

[ref45] Poczwardowski A. Lamphere B. Allen K. Marican R. Haberl P. (2019). The 5C’s model of successful partnerships in elite beach volleyball dyads. J. Appl. Sport Psychol. 32, 1–38. doi: 10.1080/10413200.2019.1573205

[ref46] Podsakoff P. M. MacKenzie S. B. Lee J. Y. Podsakoff N. P. (2003). Common method biases in behavioral research: a critical review of the literature and recommended remedies. J. Appl. Psychol. 88, 879–903. doi: 10.1037/0021-9010.88.5.879, PMID: 14516251

[ref47] Reisman J. M. (1981). “Adult friendships” in Personal relationships 2: Developing personal relationships. eds. Duck S. Gilmour R. (New York, NY: Academic Press), 205–230.

[ref48] Remelcˇ J. Vucˇković G. James N. Leskošek B. (2019). Reliability of judging in dance sport. Front. Psychol. 10:1001. doi: 10.3389/fpsyg.2019.01001, PMID: 31133935 PMC6524706

[ref49] Riemer H. A. Chelladurai P. (1998). Development of the athlete satisfaction questionnaire(ASQ). J. Sport Exerc. Psychol. 20, 127–156. doi: 10.1123/jsep.20.2.127

[ref50] Ryan R. M. (1995). Psychological needs and the facilitation of integrative processes. J. Pers. 63, 397–427. doi: 10.1111/j.1467-6494.1995.tb00501.x 7562360

[ref51] Sang H. P. (2014). Difficulty of imagining Others' behaviors and expectations about future interactions. Soc. Psychol. Germany 45, 286–298. doi: 10.1027/1864-9335/a000175

[ref52] Sara F. (2005). The intimacy of state power: marriage, liberation, and socialist subjects in southeastern China. Am. Ethnol. 32, 312–327. doi: 10.1525/ae.2005.32.2.312

[ref53] Seligman S. D. (1999). Guanxi: grease for the wheels of China. China Bus. Rev. 26, 34–38.

[ref54] Taylor J. Ashford M. Collins D. (2022). The role of challenge in talent development: understanding impact in response to emotional disturbance. Psychology 4, 668–694. doi: 10.3390/psych4040050

[ref55] Tremayne P. Ballinger D. A. (2008). Performance enhancement for ballroom dancers: psychological perspectives. Sport Psychol. 22, 90–108. doi: 10.1123/tsp.22.1.90

[ref56] Valentin M. (2020). Coding sexual violence as love-choreographed heteronormative gender performances in Latin American competitive dancing. J. Gend. Stud. 29, 962–980. doi: 10.1080/09589236.2020.1823824

[ref58] Wang B. Ye L. Wu M. Feng T. Peng X. (2014). Effects of gratitude on athlete engagement: mediation of coach-athlete relationship. J. Beijing Sport Univ. 37, 85–90. doi: 10.19582/j.cnki.11-3785/g8.2014.09.014

[ref57] Wang P. H. (2018). Dynamic dual relationship between intimate & professional— Take ballroom dance for example. Hsinchu: Institute of Mass Communication Hsuan Chuang University, 2–53.

[ref59] Wiesława P. Robert P. Walery Z. (2013). Rating size of selected characteristics and indicators of construction somatic dancers against them partners in the couples dancing, a specific choice of the standard style of dancing couples dance sports. J. Health Sci. 104, 1200–1205. doi: 10.3852/11-409

[ref9001] Wulff H. (1998). Ballet across Borders: Career and Culture in the World of Dancers. (Oxford: Berg).

[ref60] Yang E. S. (2015). Reverification of dance sport partnership scale applying Rasch model. Korea J Sport 13, 347–358.

[ref61] Yang E. S. (2016). The effects of passion on dancesport partnership. J. Korea Entertain. Industry Assoc. 10, 171–178. doi: 10.21184/jkeia.2016.06.10.3.171

[ref62] Ye Lv. (2014). The impacting factors and mechanism of engagement. (Wuhan, China: Central China Normal University).

[ref63] Ye L. Wang B. Liu Z. J. Wu Y. Y. Dong L. S. (2016). The effect of coach-athlete relationship on sport performance satisfaction—serial multiple mediating effects of hope and athlete engagement. China Sport Sci. 36, 40–48. doi: 10.16469/j.css.201607005

[ref9002] Yoshida Y. Bizokas A. Demidova K. Nakai S. Nakai R. Nishimura T. (2020). Partnering effects on joint motion range and step length in the competitive waltz dancers. J Dance Med Sci. 24, 168–174. doi: 10.12678/1089-313X.24.4.168 33218370

